# Patterns of animal rabies in the Nizhny Novgorod region of Russia (2012–2022): the analysis of risk factors

**DOI:** 10.3389/fvets.2024.1440408

**Published:** 2024-10-22

**Authors:** Olga I. Zakharova, Elena A. Liskova

**Affiliations:** Federal Research Center for Virology and Microbiology, Nizhny Novgorod, Russia

**Keywords:** rabies virus, regression, risk factors, seasonality, spatiotemporal patterns, vaccination, Nizhny Novgorod Oblast

## Abstract

**Introduction:**

Animal rabies is a viral disease that poses a significant threat to domestic and wild animal populations, with devastating consequences for animal health and human life. Understanding and assessing the risk factors associated with the transmission and persistence of the rabies virus in wild and domestic animal populations is crucial for developing effective strategies to control and mitigate cases. Studies of the spatial and temporal distribution of rabies cases in the Nizhny Novgorod region during 2012-2022 provided epidemiological evidence of the circulation of infection between animals in the presence of vaccination. Among the wild animals in the area, red foxes play a major role in the spread of rabies, accounting for 96.4% of all wild animal cases.

**Methods:**

We used spatiotemporal cluster analysis and a negative binomial regression algorithm to study the relationships between animal rabies burden by municipality and a series of environmental and sociodemographic factors.

**Results:**

The spatiotemporal cluster analysis suggests the concentration of wild animal rabies cases in the areas of high fox population density and insufficient vaccination rates. The regression models showed satisfactory performance in explaining the observed distribution of rabies in different animals (*R*^2^ = 0.71, 0.76, and 0.79 in the models for wild, domestic and all animals respectively), with rabies vaccination coverage and fox population density being among the main risk factors.

**Conclusion:**

We believe that this study can provide valuable information for a better understanding of the geographical and temporal patterns of rabies distribution in different animal species, and will provide a basis for the development of density-dependent planning of vaccination campaigns.

## 1 Introduction

Rabies in animals (RABV) is a particularly dangerous infectious disease caused by a viral pathogen belonging to the family *Rhabdoviridae*, genus *Lyssavirus* (1–3). The causative agent of rabies is an RNA-containing, neurotropic virus known for its lethality not only to domestic and wild animals but also to humans. In countries where rabies is endemic, the true extent of the problem is frequently underestimated, with the actual number of cases in both animals and humans being greater than that reported. Such underreporting causes significant damage and economic losses. These costs include expenses for treatment, control measures, and productivity losses due to illness and death caused by the rabies virus (4, 5).

Possible risk factors for the spread of rabies among wild and domestic animals include predictors such as insufficient vaccination coverage, contact between wildlife and unvaccinated domestic animals, lack of proper waste management leading to increased exposure to infected animals, and migration of wild animals. Other potential risk factors include sociodemographic and environmental conditions (such as population density, transportation network, landscape indicators etc.), poor monitoring and control measures, and human activities that encroach on wildlife areas and disrupt natural ecosystems (6–9).

The rabies virus is spread between animals by direct contact, such as bites and scratches; indirect contact through contaminated objects; maternal transmission; the migration of animals; and high wildlife densities that allow rapid spread (10, 11). Vaccination campaigns for both domestic and wild animals are essential for control efforts, as well as public awareness to promote responsible pet ownership and the reporting of suspected rabid animals. Effective vaccination strategies and control measures can greatly reduce the transmission of the rabies virus between animals, leading to the prevention of this lethal disease (11–13).

Persistent rabies foci have been identified in the Russian Federation due to the continued circulation of the virus in wildlife, particularly in foxes, raccoon dogs, arctic foxes, wolves, jackals and other animals (14). The presence of rabies cases in the wild constitutes a problem not only for veterinary medicine but also for public health in general, and disease management, even with vaccination, becomes questionable. Because of the regional variations in wildlife habitats, the presence and density of domestic and stray dogs contributes significantly to rabies epidemics in Russia and worldwide. Companion animals and stray dogs also play a significant role in contributing to rabies outbreaks worldwide (15).

As they interact with wildlife and human populations, domestic pets can act as vectors for transmitting the rabies virus. This underscores the importance of targeted interventions and public health campaigns aimed at controlling and preventing the spread of rabies, especially in densely populated areas where human–dog interactions are more frequent and the risk of transmission is greater (16–18).

Despite conducting annual preventive vaccination campaigns, rabies continues to occur in various subjects in Russia. This highlights the importance of sustained efforts to achieve and maintain high levels of vaccination to control the spread of rabies in wild and domestic animals and reduce the risk of transmission to humans (18–20).

One of the endemic regions for rabies in Russia is the Nizhny Novgorod Oblast. Cases of rabies are registered every year.

The study of spatial and temporal trends in the distribution of rabies cases within the districts of Nizhny Novgorod Oblast is a key method for understanding the dynamics of rabies spread at the district level. Spatiotemporal analysis helps to identify not only spatial clusters of rabies cases but also high-risk geographical areas exposed to animal infection during outbreaks. By identifying these patterns and areas of vulnerability, authorities can implement targeted and effective strategies to reduce the risk of rabies transmission. The publications on rabies’ spatiotemporal analysis in Russia are virtually absent in the international databases. The existing literature mainly concerns general surveillance practices (14, 21), molecular analysis (22, 23) or specific issues of vaccination (24).

Thus, the aims of this study were (1) to reveal the spatiotemporal patterns of rabies cases emergence (particularly, in regard to fox population density) in the Nizhny Novgorod region of Russia from 2012 to 2022 and (2) to identify significant risk factors and assess the impact of vaccination on the rabies emergence in various species.

## 2 Materials and methods

### 2.1 Study area

**Figure 1:**
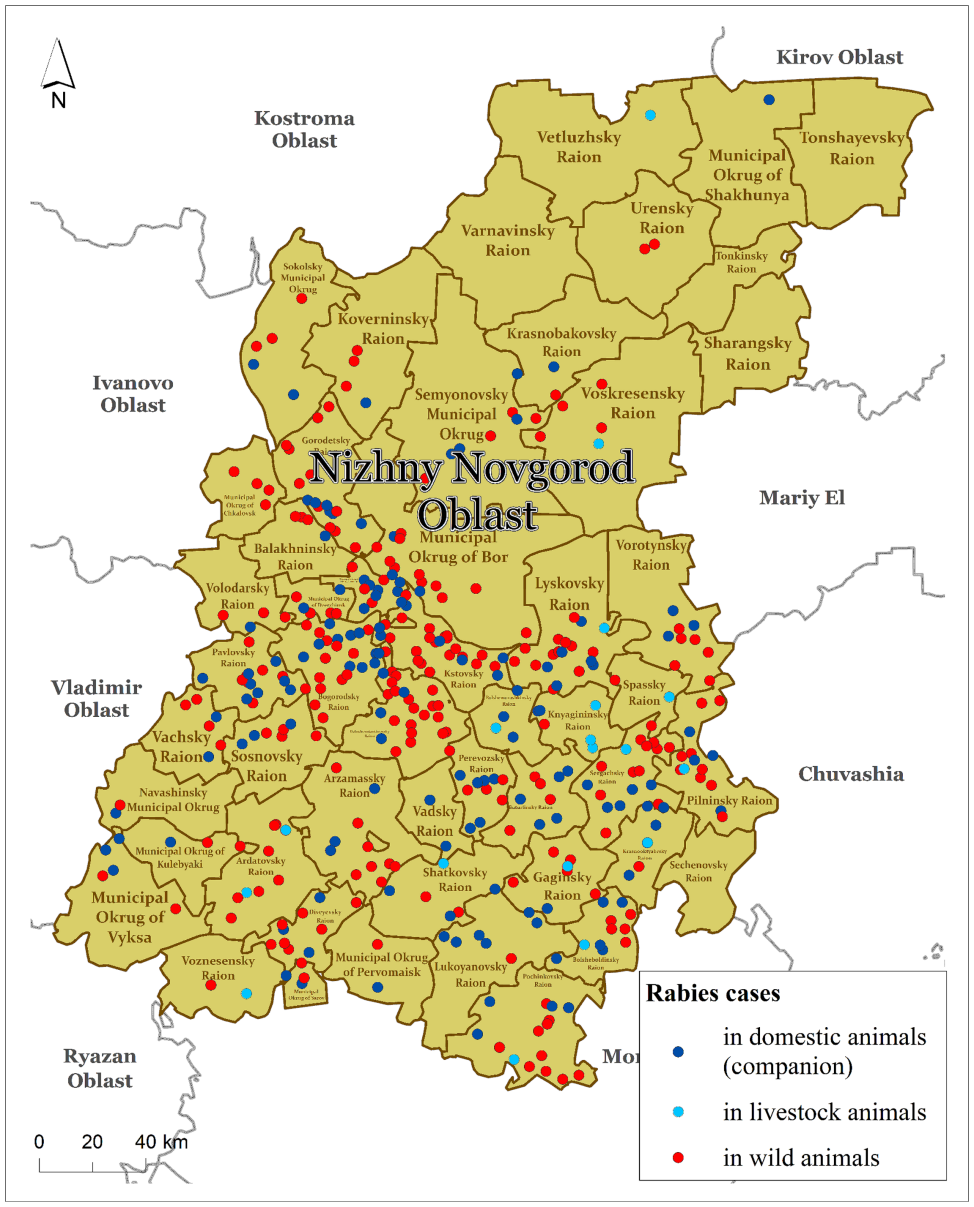
The Nizhny Novgorod Oblast of Russia and animal rabies cases, 2012–2022.

In this study, we considered the status of animal rabies in the Nizhny Novgorod Oblast of the Russian Federation from 2012 to 2022. The Nizhny Novgorod Oblast is one of the first-level administrative regions (or subjects) of the Russian Federation located in the European part of the country. The region consists of 52 s-level divisions (namely districts), which represented the model units for our study and were the minimum units of most data availability. The Nizhny Novgorod Oblast covers an area of 76.9 thousand square kilometers, or 0.31% of the total area of Russia. The climate is classified as temperate continental. The average population density is approximately 40 people per square kilometer. The region has approximately 32,000 km of rivers and more than 3,415 thousand hectares of forest, which covers 53% of the total area of the region. In the northern part of the Nizhny Novgorod Oblast, the forestry areas make up almost 80% of the total area (Figure 1).

The number of foxes in the region in early 2022 was 1,326. The highest density of foxes was observed in the southeastern part of the region. The density of foxes in the districts of Nizhny Novgorod Oblast ranges from 0.02 to 0.82 individuals per km^2^ (Supplementary material S1).

### 2.2 Rabies data

Animal rabies data from the regional veterinary laboratory’s database for the period 2012–2022 were used for this study. According to the reports of the Veterinary Committee of Nizhny Novgorod Oblast as of 31 December, 2022, 565 cases of rabies were recorded during this period.^1^ In this study, all susceptible animals were categorized as domestic (or companion), wild and livestock. As foxes were responsible for 96.4% of all cases in wild, we further consider wild animals as foxes. A rabies case is defined as the laboratory-confirmed detection of rabies virus in one or more animals associated with a geographically defined area, such as a single farm, settlement, hunting area or wildlife. Laboratory confirmation of animal rabies was carried out in the regional veterinary laboratory in accordance with the current national standard “GOST 26075–13 Animals. Methods of Laboratory Diagnostics of Rabies” (GOST 26075–2013, 2014) by the method of fluorescent antibodies (MFA) (25). The reaction was considered positive by the presence of yellow–green luminescence of granules in smears when examined under a fluorescence microscope.

### 2.3 Study design

In order to achieve the research goals, we employed the following sequence of analysis methods.

First, we performed a descriptive analysis of animal rabies data, which specifically included a calculation of the seasonality index for both domestic and wild animals as a proportion of rabies cases in a particular month out of the average total number of cases for the study period (26, 27). Additionally, we analyzed and visualized a seasonal distribution of vaccine doses for wild and domestic animals.

Second, we tested a hypothesis of the dependence of wild animals’ cases on the fox population density by comparing the spatiotemporal clusters of rabies cases in foxes detected by a Discrete Poisson SatScan model with spatiotemporal clusters of fox population density at district level detected by a Normal model.

Third, we assessed an impact of vaccination on the concentration of rabies cases by searching for population-depended spatiotemporal clusters of cases in wild animals (Discrete Poisson model) with a vaccination rate as a covariate.

Finally, we performed a regression analysis to reveal relationships between the intensity of cases in select categories of animals and a set of potential explanatory variables representing various sociodemographic and environmental conditions.

### 2.4 Potential risk factors

#### 2.4.1 Sociodemographic and landscape factors

The following district-wise variables were selected as potential explanatory factors based on the literature search: population density, number of settlements, length of main roads, number and density of susceptible animals, and total forest area (Table 1). All variables were selected on the basis of their importance in the occurrence and spread of rabies in wildlife and domestic animals, as described elsewhere (28). Data on population density and the number of livestock animals (cattle, horse, sheep) were obtained from the Federal State Statistics Service website.^2^ Data on the population of wild animals, especially foxes, were obtained from the Regional Committee for the Supervision of Hunting in the Nizhny Novgorod Oblast.^3^ Data on the number of domestic animals and information on vaccinated animals were obtained from veterinary statistical reports (see text footnote 1). The number of settlements in the Nizhny Novgorod Oblast districts was obtained from the official website of the State Statistics Service.^4^ The data on road networks and forest areas, processed from the district dataset, were obtained from the official website of Open Street Maps.^5^

**Table 1:** List of variables that were used for regression and cluster analysis of animal rabies cases the Nizhny Novgorod Oblast, Russia, 2012–2022.

Variables	Unit	Median (minimum–maximum)	VIF (variance inflation factor)
Forestry area	km^2^	495.5 (3.0–2938.0)	3.064
Percentage of forestry	%	35.7 (0.0–82.0)	2.873
Number of settlements	Unit	88.5 (2.0–1518.0)	1.124
Population density	pers/km^2^	14.3 (4.5–3,051.1)	1.377
Percentage of rural population	%	0.445 (0.01–1.00)	2.456
Summary road length	km	543.68 (21.14–1409.97)	4.833
Road density	km/km^2^	0.41 (0.29–0.61)	5.991
Rabies cases in foxes	Number	3.0 (0.0–25.0)	2.312
Rabies cases in domestic animals	Number	4.096 (0.0–15.0)	4.236
Rabies cases in wild animals	Number	6.0 (0.0–36.0)	1.675
Fox population density	Animals/km^2^	0.05 (0.012–0,893)	1.153
Vaccination coverage in wild animals	%	57,34 (32.24–82.54)	2,235
Vaccination coverage in domestic animals	%	62 (45.32–86.34)	1,326

To avoid multicollinearity, the predictors were tested for correlation using a variance inflation factor (VIF) test. Those factors with a VIF less than 5 were identified for further regression analysis (29).

#### 2.4.2 Preventive measures factors

Data on the number of doses of rabies vaccine for wildlife (30), domestic animals (31) and livestock animals (32), with indications of the name, route and method of administration according to these instructions for use, were obtained from veterinary statistical reports on routine rabies vaccination of the Veterinary Department of Nizhny Novgorod Oblast (see text footnote 1).

The vaccination coverage by districts was calculated on the basis of vaccine doses’ number and corresponding animal population number.

### 2.5 Spatiotemporal cluster analysis

#### 2.5.1 Discrete Poisson model

The clustering of disease cases and areas of increased density of susceptible animals was assessed using Kulldorff’s space–time scan statistics (33–35).

To detect high levels of spatiotemporal concentration of rabies cases, depending on the animal population size, we used a Discrete Poisson model implemented in SatScan v10.1.3 (36).

To implement a spatiotemporal model, the study area is scanned using cylindric windows of varying sizes, where the base represents space and the height represents time. The number of events observed within the windows for all size/location/time combinations compared to the number expected under a null-hypothesis. The statistic then maximizes the Poisson likelihood function across window radius, heights and starting locations to identify the most likely cluster and possible secondary clusters (37). The null hypothesis for this model is that the expected number of cases of disease in particular areas follows a Poisson distribution based on the size of the susceptible population in the same area (38–40). Additional variables that presumably affect the theoretical distribution may be added as covariates. In our case, we added a vaccination rate in wild animals. The *p*-value for all clusters was calculated via 999 Monte Carlo simulations. Clusters with *p* < 0.05 were considered to indicate statistical significance. The spatial and temporal sizes of the scanning window was set by default as 50% of the whole study population and time period, respectively. Relative risk (RR) estimates indicate that more or less expected numbers occurred in a given category. RR > 1 represents a greater than expected number of individuals in a certain category inside the spatial cluster compared to outside.

#### 2.5.2 Normal model

To identify concentration of districts with increased wildlife density, a spatial–temporal cluster analysis was conducted using a Normal probability model (41). This model for the analysis of continuous data assumes that the theoretical (expected) distribution of the study parameter is normal. The model identified clusters with statistically significant (*p* < 0.05) fox population densities that exceeded the expected values (42).

For either model, the cluster analysis returns a number of statistical metrics that include: a cluster radius, cluster duration, a ratio of observed to expected number of cases within the cluster (ODE), a Relative Risk indicating the ratio of observed rabies foci within the cluster to the expected number of cases outside the cluster, and a *p*-value indicating the statistical significance of the found cluster as compared to the null-hypothesis.

### 2.6 Regression analysis

The risk factor analysis used a negative binomial regression model, where the response variable was the total number of rabies cases during the study period by district in the population of (1) wildlife, (2) domestic (companion)—dogs and cats, and (3) in all animals. The negative binomial regression model is typically used to model count data when the variation of the response variable exceeds its mean (i.e., overdispersion) (43). The choice of the negative binomial regression model in our case was justified by the distribution of the number of rabies cases, which showed pronounced overdispersion: for cases of rabies in wild animals, the mean value was 5.37 (variance of 37.22); for cases in domestic companion animals (dogs and cats), the mean value was 4.10 (14.29); and for cases in all animals, the mean value was 9.40 (78.28). The regression models were adjusted using stepwise removal of independent variables to achieve the lowest value of the Akaike information criterion (AIC) using the stepAIC procedure. The significance of the variables was determined using Student’s t test. The *p*-value indicates statistical significance. The overall goodness of fit of the models was determined by means of R-squared. The spatial distribution of both the response variable and the model residuals was evaluated using Moran’s I test for spatial autocorrelation, which assesses the conformity of the observed spatial distribution of the analyzed variable with a hypothetical random distribution (null hypothesis). The presence of spatial autocorrelation in both the response variable and the residuals indicates unexplained clustering of the phenomenon not accounted for by the explanatory variables (44, 45).

### 2.7 Software

Statistical processing of the data was performed using the MS Office Excel application package (Microsoft, Redmond, WA, USA). Cluster analysis was performed using SaTScan v10.1.3 (42). Regression analysis was performed using R statistically-oriented software version 4.9.0.^6^ The spatial analysis and visualization of the results were carried out using ArcMap 10.8.2 and ArcGIS Pro 2.7.0 (Esri, Redlands, CA, USA).

## 3 Results

### 3.1 Descriptive analysis

**Figure 2:**
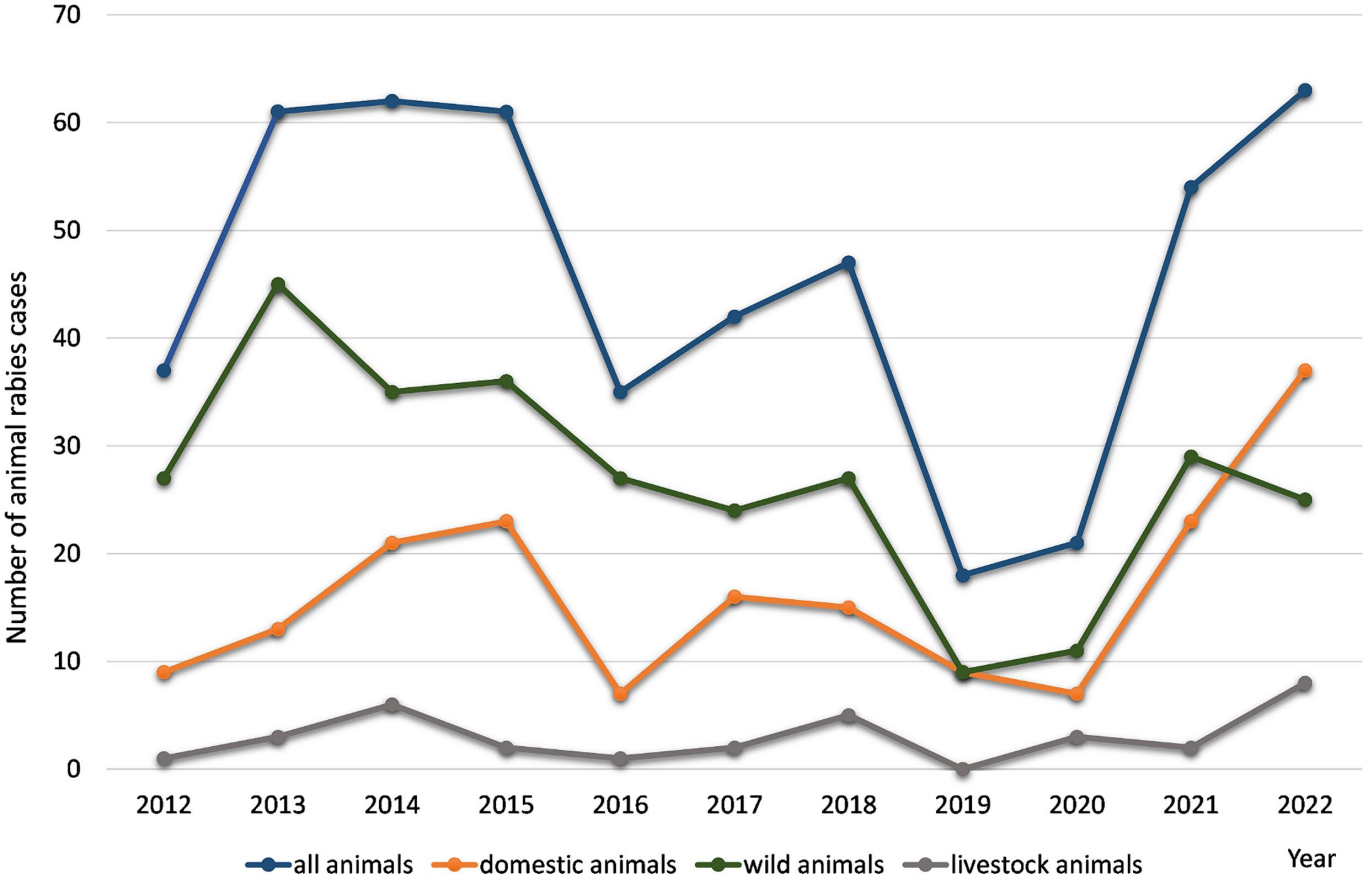
The yearly distribution of rabies cases in various animal in the Nizhny Novgorod Oblast, 2012–2022.

Based on the results of a retrospective analysis of rabies cases recorded in the districts of the Nizhny Novgorod Oblast from 2012 to 2022, it was found that the maximum number of infected animals was observed in 2013–2015 and 2021–2022, and ranged from 61 to 65 cases per year. The minimum number of cases occurred in 2019 and 2020, with 18 and 21 cases, respectively (Figure 2).

Geographically, all rabies cases were distributed within almost all districts, covering 88.46% of the total study area. At the beginning of 2012, rabies cases were detected in animals in central districts of the Nizhny Novgorod region. In 2014, there was an expansion of the rabies area to the southern districts of the region (42 cases). In 2016, rabies cases were registered in the north districts of the region. The decrease in the number of cases recorded in 2019–2020 to 18–21 cases was mainly observed in domestic companion animals (dogs and cats). In 2021 and 2022, there was an increase in the number of registered cases among wild and domestic (companion) animals, which may be related to an ineffective rabies vaccination campaign.

In the Nizhny Novgorod region, the main animal species involved in the transmission of the rabies virus (2012–2022, *n* = 565) were wild animals (56.7 ± 10.7%). The second most common infected population was domestic companion animals - dogs and cats (24.2 ± 7.2% and 11.8 ± 2.5%, respectively). Livestock animals accounted for 4.5 ± 1.5% of the total number of rabies cases. Another feature of the entire study period is the consistently high incidence of rabies virus infection in foxes, which accounts for more than 96.4% of cases in wild animals.

**Figure 3:**
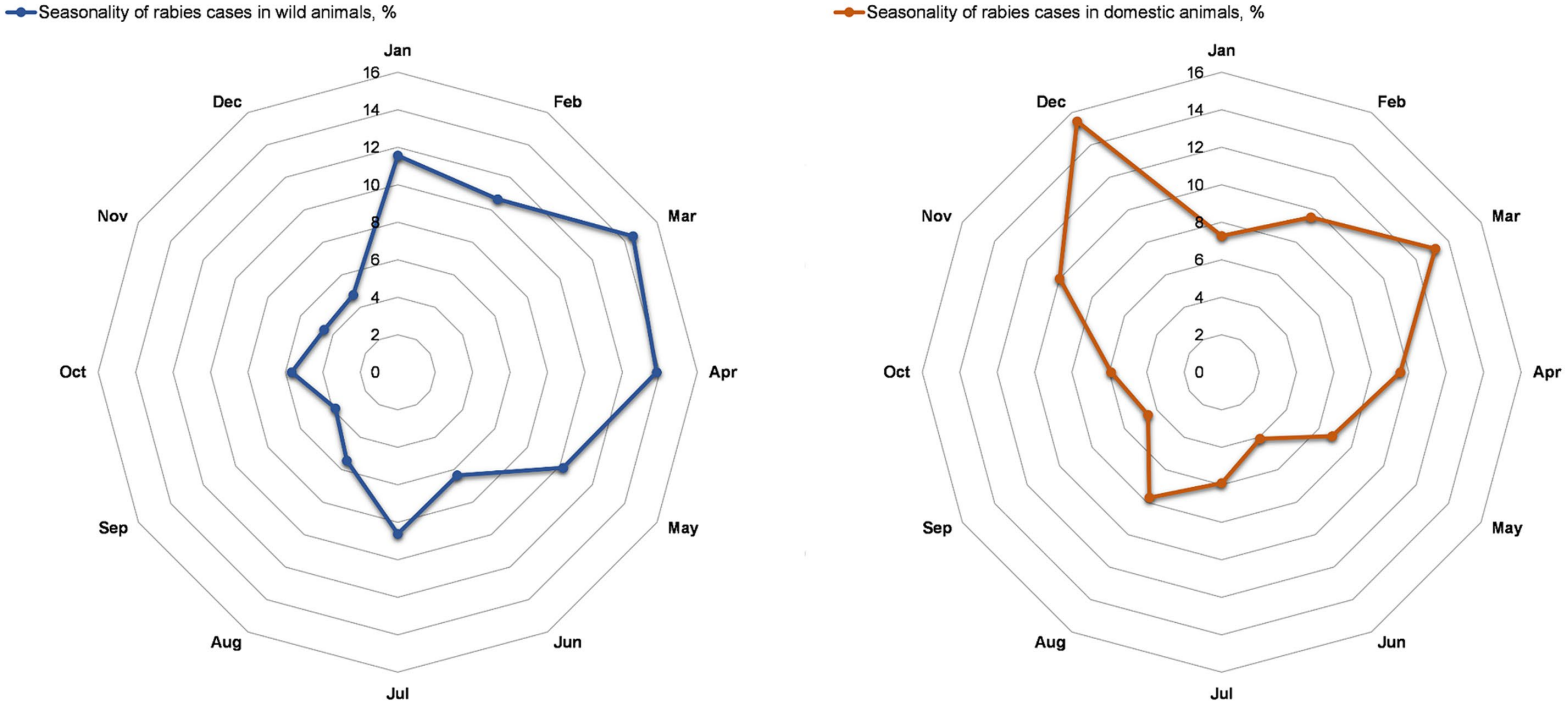
Seasonality of rabies cases in wild animals **(A)** and domestic (companion) animals **(B)** in the Nizhny Novgorod Oblast, 2012–2022.

The seasonality of registered rabies cases in wild animals showed the first peak in March–May (12.4–9.9%) and the second peak in January (12.0%) (Figure 3A). Seasonal peaks of rabies cases in domestic animals were observed in March and December, with 13.7 and 15.8%, respectively (Figure 3B).

**Figure 4:**
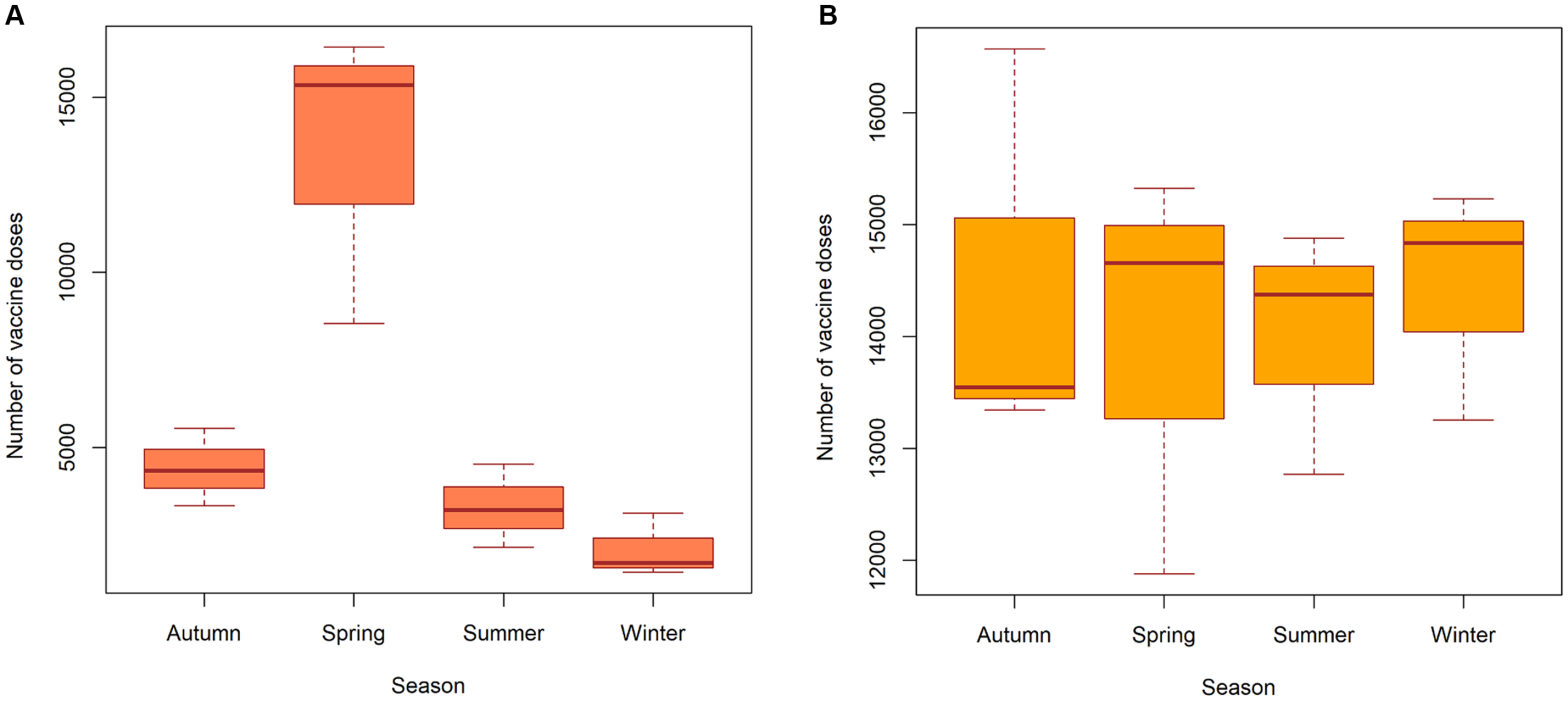
Seasonal distribution of vaccine doses in wild animals **(A)** and domestic (companion) animals **(B)** in the Nizhny Novgorod Oblast, 2012–2022.

The distribution of vaccination doses administered to wild animals, as shown in Figure 4A, indicates that the activity was carried out during the spring period with maximum coverage of the susceptible population. Vaccination of wild animals was also conducted in the autumn and summer seasons but to a lesser extent than in the spring. The distribution of vaccination doses used for domestic animals, as shown in Figure 4B, indicates that the activity was carried out in roughly equal amounts throughout all seasons of the year, in line with the vaccination plan for domestic companion animals. Information on the vaccine coverage of animals (wild, domestic and livestock animals) by districts of Nizhny Novgorod Oblast is available in the Supplementary material S2.

### 3.2 Spatiotemporal analysis

#### 3.2.1 Comparing spatiotemporal clusters of rabies cases in wild animals with clusters of fox population density

Spatiotemporal analysis of rabies foci among wild animals in the districts of the Nizhny Novgorod region using the Discrete Poisson model revealed a total of four statistically significant clusters with different time periods of formation from 2 to 5 years.

Among these clusters, cluster #4 with a radius of 21.62 km and a duration of 5 years, had the highest relative risk of disease spread (38.35) and ODE (27.47).

Clusters #1 and #2 are located in geographically different areas of the region and were short lived, lasting from 3 to 4 years. The relative risk (RR) in the clusters ranged from 13.344 to 23.362. The geographical areas within the formed clusters could be classified as high-risk areas for rabies cases among wild animals because local areas with fox density in cluster #1 reached to 0.82 individuals per km^2^, and those in the second cluster reached to 0.50 individuals per km^2^.

Cluster analysis of the fox population density using a normal model revealed only one statistically significant cluster (namely cluster #5) with a radius of 95.81 km located in the southern part of the region and covering 29 districts. The main characteristics of the identified clusters are presented in Table 2.

**Table 2:** Characteristics of clusters of rabies cases in wild animals (Discrete Poisson model) and fox population density (Normal model), 2012–2022.

Model	Number of cluster (corresponds to the map in Figure 5)	Cluster radius (km)	Start year	End year	ODE	Relative risk (RR)	*p*-value
Clusters of rabies cases in wildlife (Discrete Poisson model)	1	43.176	2013	2016	12.960	23.362	0.000
	2	40.942	2012	2014	8.102	13.344	0.000
	3	0.000	2014	2015	6.620	6.799	0.003
	4	21.620	2016	2020	27.47	38.345	0.000
Fox population density clusters (Normal model)	5	95.807	2012	2016	30.944	–	0.000

ODE—observed/expected (the ratio of the observed number of rabies cases to the expected number within a cluster, provided the distribution corresponds to the null hypothesis).

Relative Risk (RR)—the relative risk value shows the ratio of observed rabies foci within the cluster to the expected number of cases outside the cluster.

**Figure 5:**
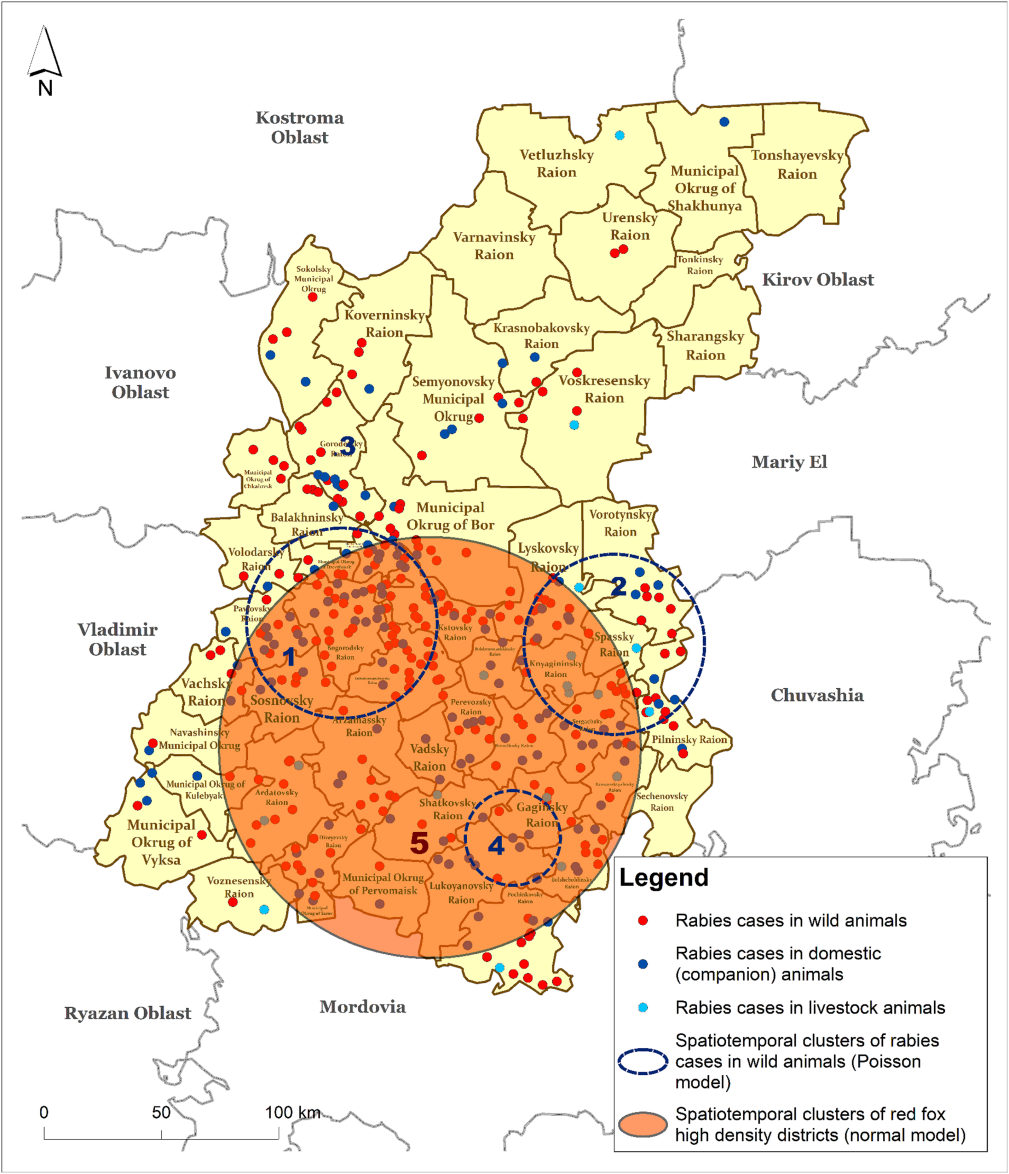
Spatiotemporal clusters of rabies cases in wild animals (Poisson model) vs. clusters of fox density (Normal model) in the Nizhny Novgorod Oblast, 2012–2022.

Clusters #1 and #2 partially overlap with the red fox population density cluster. Full spatial and temporal (2016) coincidence with the cluster of fox density is represented by cluster #4, with an ODE of 27.47. The districts included in cluster #4—Gaginsky, Lukoyanovsky, Shatkovsky, Bolshieboldinsky, and Pochinkovsky—coinciding with cluster #5 identified by the normal probability method represent high-risk areas for the spread of rabies among wild animals, particularly foxes (Figure 5).

#### 3.2.2 Spatiotemporal cluster analysis of rabies in wild animals with regard to a vaccination rate

**Figure 6:**
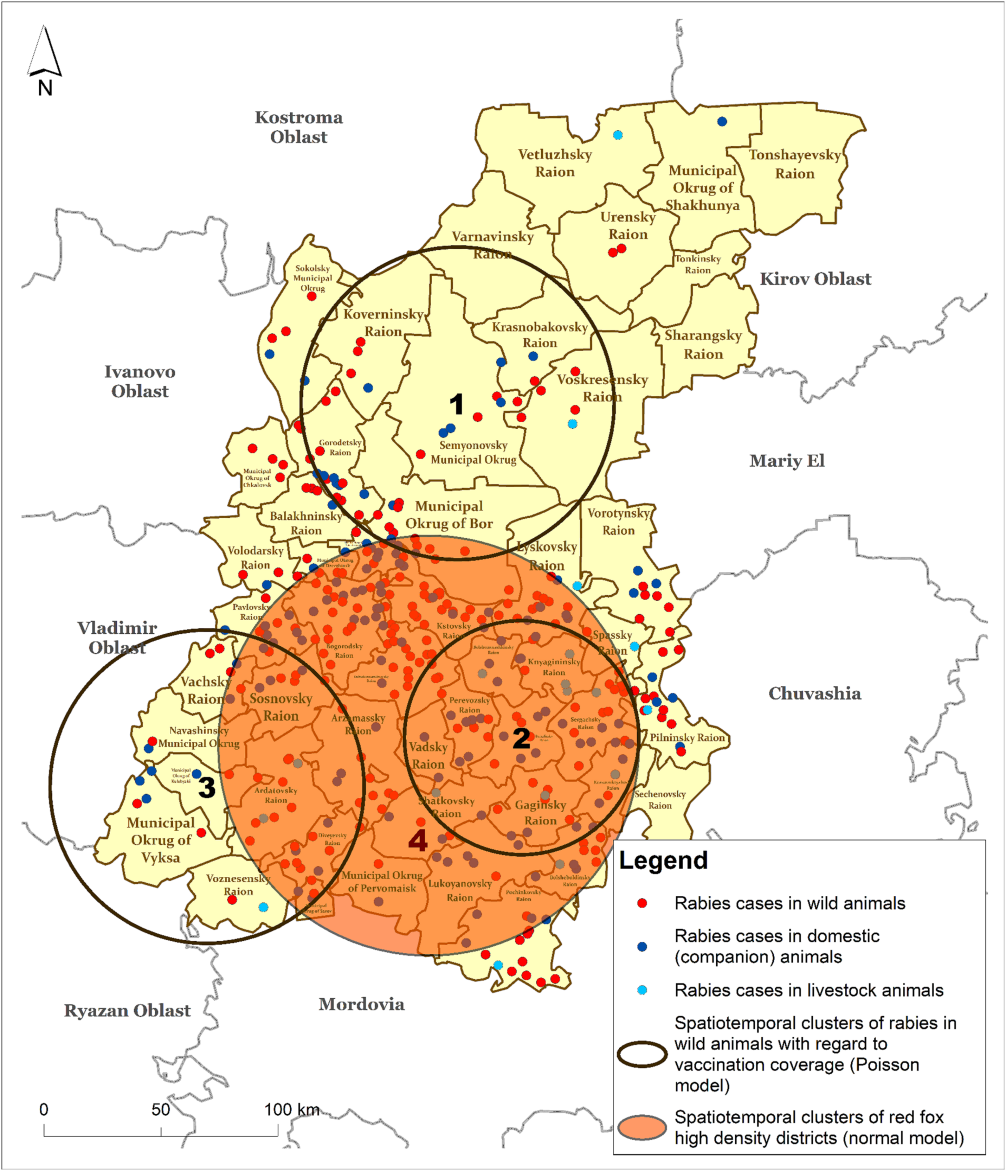
Spatiotemporal clusters of rabies cases in wild animals with respect to the vaccination rate (Poisson model) vs. clusters of fox density (Normal model) in the Nizhny Novgorod Oblast, 2012–2022.

The results of the cluster analysis of rabies cases among wild animals, obtained using a Discrete Poisson model based on the number foxes with a covariate representing a number of vaccinated individuals, revealed three statistically significant clusters. In this case, we were looking for clusters of low values, that is, clusters of rabies cases in wild animals based on the population density covered by vaccination. Clusters 1–3 of rabies in wild animals shown in Figure 6 were formed due to insufficient vaccination coverage against rabies in the wild animal population. Cluster numbers 2 and 3, with ODE values of 0.183 and 0.179, respectively, represent the highest risk of rabies spread in the wild animal population (Table 3).

**Table 3:** Characteristics of clusters of rabies cases in wild animals (Discrete Poisson model) with regard to the vaccination rate in wildlife (Normal model), 2012–2022.

Model	Number of cluster (corresponds to the map in Figure 6)	Cluster radius (km)	Start year	End year	Relative risk	ODE	*p*-value
Clusters of rabies cases in wildlife (Discrete Poisson model)	1	70.83	2019	2022	0.093	0.104	0.000
	2	53.42	2015	2020	0.166	0.183	0.000
	3	71.57	2020	2022	0.165	0.179	0.003
Fox population density clusters (Normal model)	4	95.807	2012	2016	–	30.944	0.000

LLR—(log-likelihood ratio)—a statistical test used to test constraints on the parameters of statistical models estimated from sample data.

A full spatial and temporal coincidence of cluster #2 of rabies cases among wild animals with cluster #4 of increased fox population density was found.

### 3.3 Regression analysis of risk factors for rabies in wild animals

The regression model applied to the number of rabies cases in wild animals confirmed the rabies vaccination factor (with a negative regression coefficient) and a fox population density to be statistically significant determinants. The model demonstrated a good fit with *R*^2^ = 0.71 (Table 4).

**Table 4:** Statistical metrics of the regression model to determine risk factors of rabies in the population of wild animals in the Nizhny Novgorod Oblast, 2012–2022.

Variable	Regression coefficient	Standard error	CI (95%)	*p*-value
Intercept	−0.999	0.144	−1.284–(−0.719)	0.000
Fox population density, animals/km^2^	1.193	0.289	0.558–2.233	0.000
Percentage of forestry areas, %	0.547	0.012	0.032–0.856	0.000
Vaccination rate in wild animals, %	−1.798	0.559	−2.947–(−0.534)	0.001

CI, confidence interval.

The Moran’s I test statistics of the regression residuals was 1.0262 with the *p*-value of 0.617. Since the *p*-value is greater than 0.05, we can accept the null hypothesis and conclude that there is no autocorrelation in the residuals of this regression model.

### 3.4 Regression analysis of risk factors for rabies in domestic (companion) animals

The most important predictors for the spread of rabies among pets in the Nizhny Novgorod region were the number of rabies cases in foxes, as well as the vaccination rates among wildlife, domestic animals, and livestock, as revealed by the regression model results (Table 5).

**Table 5:** Statistical metrics of the regression model to determine risk factors of rabies in the population of domestic (companion) animals in the Nizhny Novgorod Oblast, 2012–2022.

Variable	Regression coefficient	Standard Error	CI (95%)	*p*-value
Intercept	1.020	0.134	0.755–1.279	0.000
Rabies cases in foxes, number	0.184	0.034	0.049–0.284	0.000
Vaccination rate in domestic animal, %	−1.03	0.43	−0.78–(−0.45)	0.000
Vaccination rate in wild animals, %	−1.08	−0.234	−0.123–0.25	0.000

CI, confidence interval.

The *R*^2^ was 0.76. The results obtained for autocorrelation using the Moran’s I test showed that the test statistic was 1.932, and the *p*-value was 0.204 suggesting no autocorrelation of the regression residuals.

### 3.5 Regression analysis results of risk factors for rabies in different animals

The results of the regression model for rabies cases among all animals identified the most important risk factors, which are presented in Table 6.

**Table 6:** Statistical metrics of the regression model to determine risk factors of rabies in the population of all animals in the Nizhny Novgorod Oblast, 2012–2022.

Variable	Regression coefficient	Standard error	CI (95%)	*p*-value
Intercept	1.083	0.144	0.798–1.361	0.005
Fox population density, animals/km^2^	1.278	0.031	0.569–1.414	0.000
Population density, person/km^2^	0.026	0.008	0.017–0.435	0.002
Number of settlements	0.047	0.013	0.023–0.079	0.014
Percentage of forestry areas, %	0.014	0.006	0.011–0.049	0.039
Vaccination rate in wild animals, %	−1.042	0.023	−0.580–0.234	0.050

CI, confidence interval.

**Figure 7:**
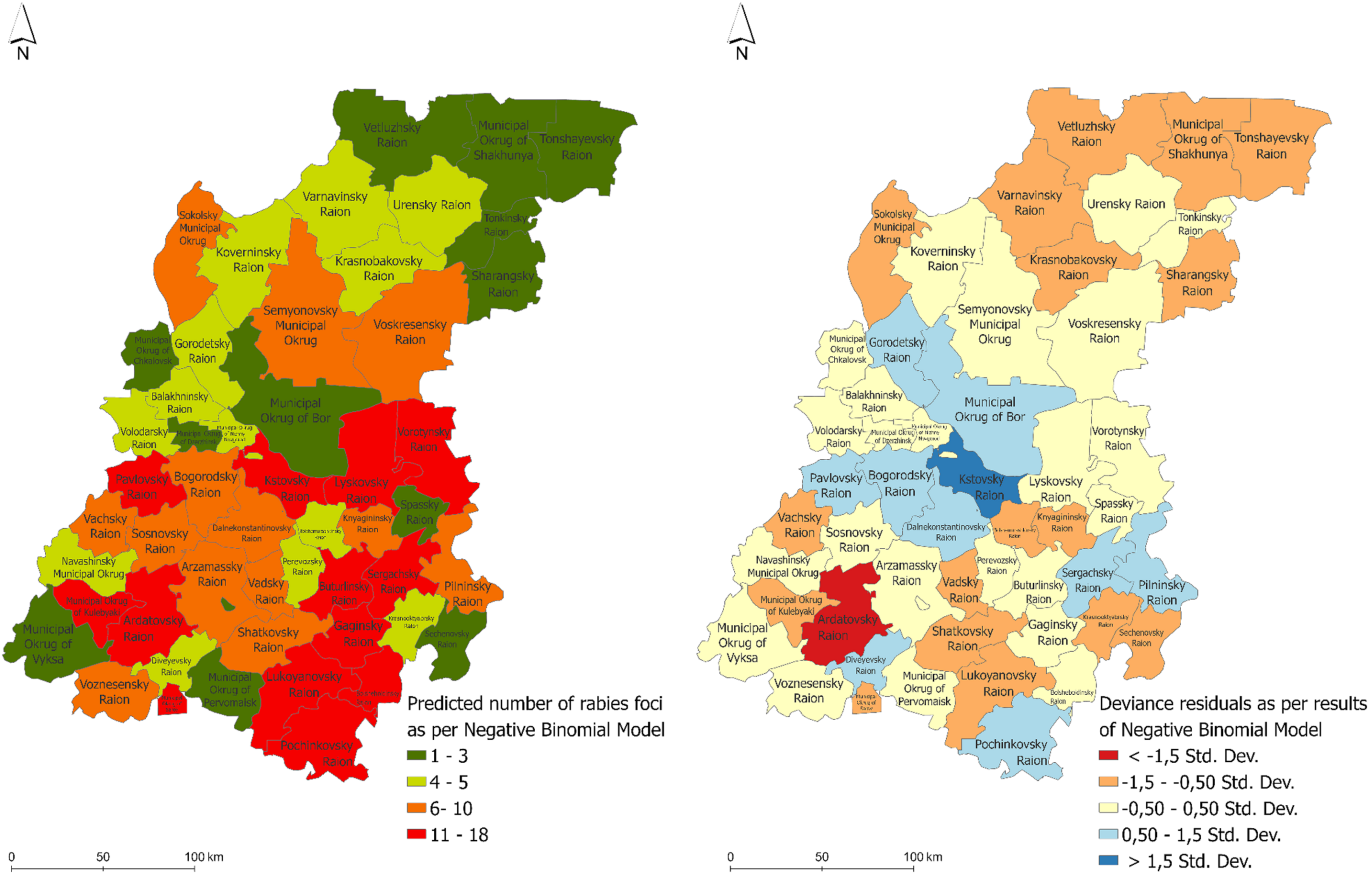
The predicted number of rabies cases in various animals in the Nizhny Novgorod Oblast as estimated by the negative binomial regression model (left) and the distribution of the associated regression residuals (right).

The maps of the predicted number of rabies cases in allanimal species and the distribution of the regression residuals are presented in Figure 7. The areas at high risk for the rabies emergence included the following districts: the Pavlovsky, Lyskovsky, Vorotynsky, Kstovsky, Buturlinsky, Sergachsky, Gaginsky, Kulebaksky, Ardatovsky, Lukoyanovsky, and Pochinkovsky districts. This high risk area is mainly located in the southern part of the Nizhny Novgorod region.

The coefficient of determination of the regression model (*R*^2^) was 0.79. The Moran’s I index of the regression residuals was 0.096, with the *p*-value of 0.204 that suggests no spatial autocorrelation in the model residuals.

## 4 Discussion

Despite the availability of many safe and effective vaccines worldwide, animal rabies remains a socially significant zoonosis, threating animal and human life and health. The vaccination of animals is essential for preventing the spread of rabies. Studies continue to demonstrate the importance of vaccinating animals, especially pets in close contact with humans, to prevent the disease in animals and reduce the risk of transmission (46–48).

Incomplete data on rabies cases make it difficult to assess the health status of animals and to understand the contribution of various risk factors to the transmission of the rabies virus, which consequently leads to an underestimation of the importance of the problem (49, 50).

One of the tasks of the study of rabies in animals in the Nizhny Novgorod region was to determine the spatial and temporal characteristics of the disease spread in connection with the identification of significant risk factors, including vaccination as an important predictor in the containment of rabies.

This study revealed that rabies is widespread throughout most districts of the Nizhny Novgorod region, indicating a persistence of the disease. Analysis of rabies cases in different animal species revealed that 46 out of 52 districts (88.46%) were affected, particularly in the southern and southeastern areas. These districts have ongoing rabies cases. The majority of cases in wild animals (over 96.4%) were reported in foxes. This highlights the red fox as the main reservoir of the disease in the most districts of the Nizhny Novgorod Region (51, 52). The frequent detection of rabies in wildlife is likely linked to the high density of foxes and the high density of domestic animals (dogs and cats) (53).

Studying the seasonal patterns of rabies outbreaks in all animal species to identify peak seasons is crucial for planning an effective vaccination campaign to protect vulnerable animal populations. This study identified two peaks in the incidence of rabies in wild animals in Nizhny Novgorod Region. The first peak of rabies cases was observed from March to May (12.4–9.9%), and the second peak was in the winter season, from December to January (9.6–9.0%). The rabies peak from March to May can be explained by an increase in the potential number of wild animals (reservoirs), specifically foxes. Understanding the biology, ecology, breeding and migration patterns of wild animals exposed to the rabies virus can improve our understanding of seasonal animal morbidity patterns. The breeding of domestic animals does not follow a specific seasonal pattern, and there is a possibility that it is influenced by particular environmental conditions that focus on the process at a particular time of year.

Vaccination campaigns involving domestic (companion) animals were carried out throughout the year in the Nizhny Novgorod region. However, under certain conditions, there is a seasonal aspect in the incidence of rabies in pets, which may be attributed to the breeding of stray pets in urban and suburban areas.

Other possible reasons for the seasonal trends in the incidence of rabies in animals could be the characteristics of natural conditions, the probability of contact due to migration and/or breeding, and the availability of food (54, 55).

Spatiotemporal analysis of rabies in wildlife in the Nizhny Novgorod region from 2012 to 2022 identified high fox population density as one of the factors in the spread of infection. Clusters of rabies cases were identified in districts with a high density of foxes. High density of host animals helps spreading rabies by increasing the likelihood of infected and healthy animals coming into contact (56, 57). Clusters of rabies cases have been reported in districts where fox densities are stable. This is particularly the case in the central and southeastern districts. Four significant clusters of rabies were identified in districts with fox densities between 0.02 and 0.80 foxes/km^2^.

Vaccination coverage ranges from 10 to 55% in the area of the rabies cases’ cluster #4, which overlaps with cluster #5 of high fox population density. The factors contributing to insufficient vaccination coverage may include low-quality bait and a vaccine distribution that does not follow animal migration routes, as well as inadequate planning that does not take into account the distribution of fox density.

Variations in vaccination coverage of wild and domestic animals between districts may contribute to the formation of clusters of rabies cases observed in this study. The clustering of rabies cases with low vaccination rates and sustainable fox densities suggests that rabies is endemic to these areas despite routine vaccination measures (24, 58).

The analysis of risk factors for the spread of rabies in wild and domestic animals in the Nizhny Novgorod region included landscape and sociodemographic predictors, as well as a determinant of animal prevention measures—vaccination. The main predictors of the spread of rabies among all animals in the Nizhny Novgorod region were the density of fox population, the number of settlements, the vaccination coverage of wild animals, the human population density and the percentage of forest cover. This particularly underlines the role of foxes as a leading factor of rabies persistence and spread in the region, and also suggests the virus transmission from wild to domestic populations in the densely populated areas (59, 60).

The significant regression coefficient for fox population density was found to be 1.278 (0.569–1.414). This factor, which is crucial for the spread of infection among animals, is likely due to increased contact among susceptible animals. The likelihood of transmitting the virus increases, particularly in close contact, due to the limited geographic location of fox habitats and anthropogenic influences on changing the habitat of wild animals and density-dependent contact ratios (61, 62).

Forested areas in the Nizhny Novgorod region provide habitat for a wide range of wildlife species, including potential animal reservoirs for the rabies virus. The percentage of forest can create barriers to control and make it difficult to access and vaccinate wild animals, potentially facilitating the persistence and spread of rabies in wild animal populations (6, 21). Regression analysis of risk factors, including vaccination, revealed that reported cases of rabies in wild and domestic animals in the Nizhny Novgorod region were related to the number of vaccine doses administered. The proportion of vaccinated animals in a wild animal population can have an impact on the spread of rabies to other animals. A higher vaccination rate results in a protective barrier to immunity and a break in the chain of transmission of rabies within the animal population. It should be noted that our study was based on the number of administered vaccine doses rather than on the direct indicators of protective immunity in animals. A more detailed study of the percentage of wild animals, including foxes, vaccinated against rabies (monitoring), was not possible due to the lack of laboratory test data confirming the presence of the rabies vaccine in the bodies of wild animals.

A regression analysis with regard to the rabies in domestic animals revealed the dependence on the number of rabies cases in wildlife, and on the vaccination rates in both domestic and wild population. This finding contributes to the understanding of rabies in domestic animals as a secondary process closely related to the epidemic situation in wild animals (7, 63).

The risk map consistently identified the southern and southeastern regions of Nizhny Novgorod and certain districts in the central region, represented by the Kstovsky and Bogorodsky districts, as high-risk zones for rabies in wild animals. The northern territories of the region did not show such a risk in wild animals, probably due to the relatively low susceptible population density.

Ongoing cases of rabies in wildlife in the Nizhny Novgorod region are likely due to vaccine distribution. This may not cover the entire wildlife population. Another possible reason may be that existing counting methods may be not accurate enough to estimate the number of wildlife, making it difficult to assess the distribution of carnivore species. This runs the risk of under vaccinating wildlife throughout the affected districts. Areas where more vaccine doses are demanded can be identified by estimating the density of wild animals per district. Using regression modeling, a risk map will help to plan effective preventive vaccination strategies for wildlife and pets. The spread of rabies can be prevented through the regular and widespread vaccination of both wild and domestic animals.

Proper monitoring of animals and strict compliance with veterinary regulations can reduce new cases of the disease and disrupt the cycle of the rabies virus in animal populations.

## 5 Conclusion

Our research findings on identifying spatiotemporal patterns of rabies among various animals in the districts of the Nizhny Novgorod region enhance the understanding of geographical and seasonal aspects of the disease manifestation in the region. Red fox density and vaccination coverage of both wild and domestic animals populations were found to be the most significant risk factors related to rabies intensity in the study region. This confirms the leading role of wildlife as a main reservoir of rabies and underlines the need to accurately consider the fox population distribution in order to provide an effective planning of density-dependent vaccination campaigns. We believe that this study can provide valuable information for a better understanding of the geographical and temporal patterns of rabies spread among different animal species based on vaccination capabilities. It can also assist in developing veterinary service policies on vaccinating wildlife and domestic animals and in creating a unified strategy to eradicate rabies in the region. Efforts should focus on understanding the transmission of the virus between these animals, optimizing the geographical distribution of vaccine coverage, and implementing other risk reduction measures.

## Data Availability

The original contributions presented in the study are included in the article/Supplementary material, further inquiries can be directed to the corresponding author.

## Footnotes

^1^https://vetnadzor.nobl.ru/

^2^https://eng.rosstat.gov.ru/

^3^https://ohotnadzor.government-nnov.ru/?id=87104

^4^https://rosstat.gov.ru/

^5^https://www.openstreetmap.org/#map=3/69.62/-74.90

^6^https://cran.r-project.org/bin/windows/base
