# Supplementary material for: Patterns of animal rabies in the Nizhny Novgorod region of Russia (2012–2022): the analysis of risk factors
Source: Front Vet Sci. 2024 Oct 22;11:1440408. doi: 10.3389/fvets.2024.1440408 (PMC11536352; doi:10.3389/fvets.2024.1440408)

Supplementary Material

1 Supplementary Figures

Supplementary Figure 1. Fox population density in the Nizhny Novgorod Oblast, 2012-2022, head/km^2

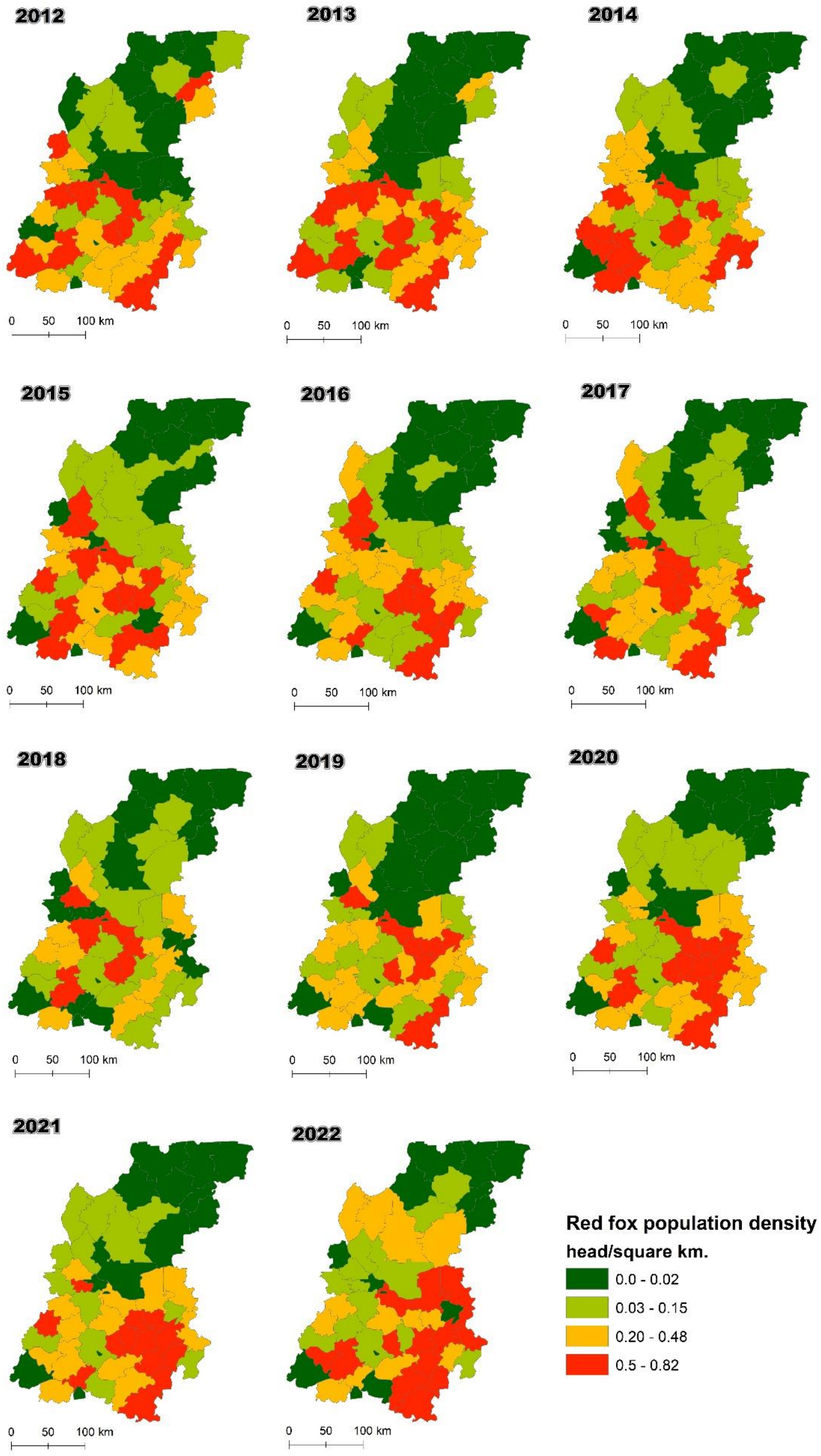
2012
2013
2014
2015
2016
2017
2018
2019
2020
2021
2022
0 50 100 km
Red fox population density
head/square km.
0.0 - 0.02
0.03 - 0.15
0.20 - 0.48
0.5 - 0.82

Supplementary Figure 2. Percentage of the wild animal vaccination coverage in the Nizhny Novgorod Oblast from 2012 to 2022, %

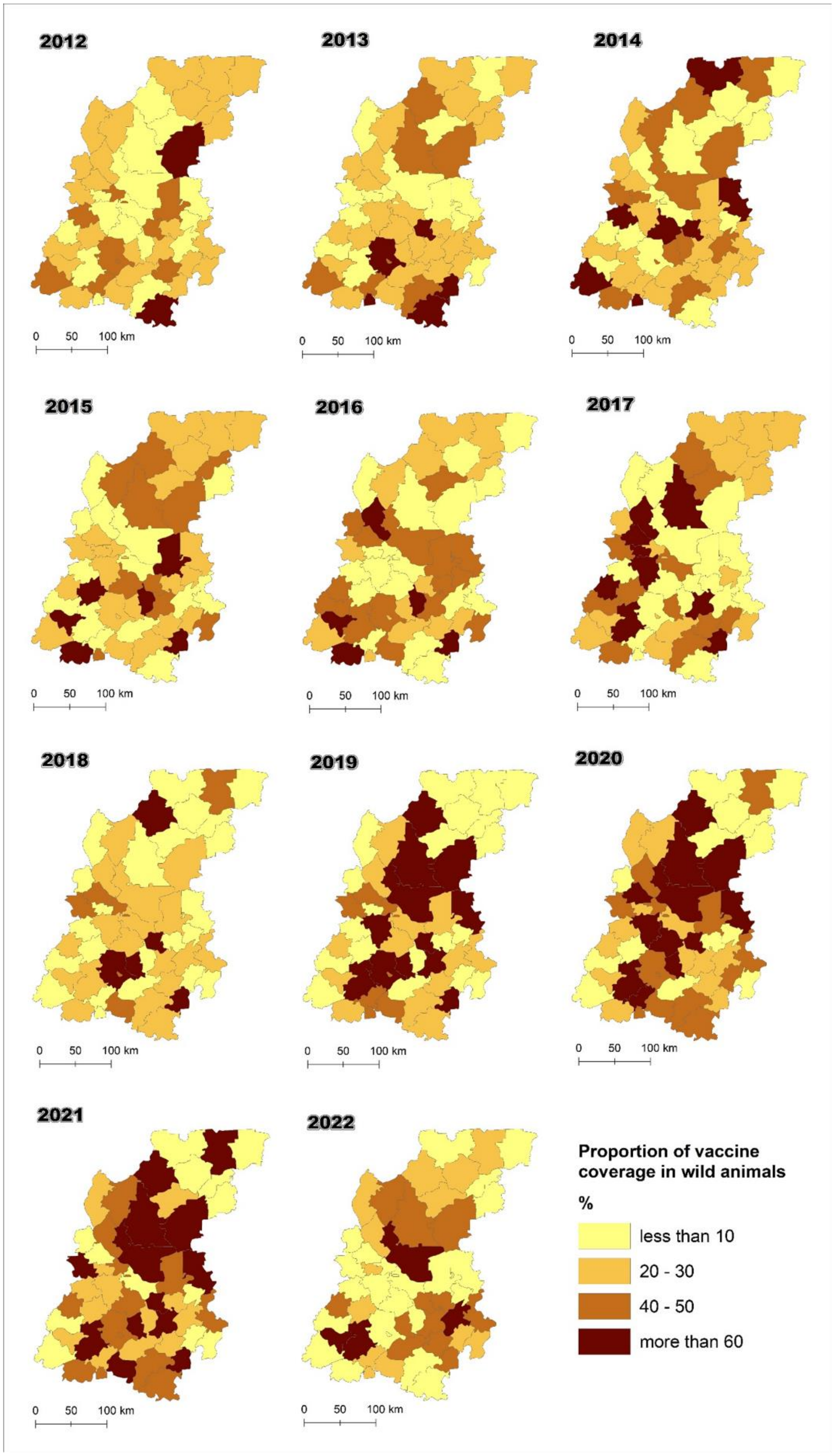

Supplement: Supplementary file 1 [file Image_1.pdf]
